# Supplementary material for: Video swin-CLSTM transformer: Enhancing human action recognition with optical flow and long-term dependencies
Source: PLoS One. 2025 Jul 7;20(7):e0327717. doi: 10.1371/journal.pone.0327717 (PMC12233284; doi:10.1371/journal.pone.0327717)
Supplement: Attachment — Submitted filename: Response to Reviewers.docx [file pone.0327717.s002.docx]

**Response to Reviewers**

Dear Editors and Reviewers:

Thanks for your letter and for the reviewers’ comments concerning our manuscript entitled “Video Swin-CLSTM Transformer: Enhancing Human Action Recognition with Optical Flow and Long-Term Dependencies” (**PONE-D-24-45158**). We sincerely acknowledge the reviewers for their constructive comments that are much helpful to improve the quality of our manuscript. We have addressed all the comments point by point in the response and revised the manuscript accordingly. We hope that the revised manuscript now meets your expectations and is accepted for publication.

**Journal requirements:**

When submitting your revision, we need you to address these additional requirements.

1. Please ensure that your manuscript meets PLOS ONE's style requirements, including those for file naming.

Response: Thanks for your kind reminder. We have ensured that our manuscript meets PLOS ONE's style requirements.

2. Please note that PLOS ONE has specific guidelines on code sharing for submissions in which author-generated code underpins the findings in the manuscript. In these cases, we expect all author-generated code to be made available without restrictions upon publication of the work. Please review our guidelines at [https://journals.plos.org/ploson
-e/s/materials-and-software-sharing#loc-sharing-code](https://journals.plos.org/ploson-e/s/materials-and-software-sharing#loc-sharing-code) and ensure that your code is shared in a way that follows best practice and facilitates reproducibility and reuse.

Response: Thank you for the kind reminder. Our core experimental code is open-sourced on GitHub and accessible to all users at the link: [[https://github.com/cherrycsw/
Video_Swin_CLSTM](https://github.com/cherrycsw/Video_Swin_CLSTM)].

3. Thank you for stating in your Funding Statement: [This work was supported by the Hubei Province Key Research and Development Special Project of Science and Technology Innovation Plan (No. 2023BAB087), the Wuhan Key Research and Development Projects (No. 2023010402010614), the Central Government Guides Local Funds for Science and Technology Development (No. ZYYD2024QY08) and the Wuhan Knowledge Innovation Special Dawn Project (No. 2023010201020465). Further support was provided by the open competition project for selecting the best candidates, Wuhan East Lake High-tech Development Zone (No. 2024KJB328) and the Fund for Research Platform of South-Central Minzu University (No. CZQ24011).].Please provide an amended statement that declares *all* the funding or sources of support (whether external or internal to your organization) received during this study, as detailed online in our guide for authors at http://journals.plos.org/plosone
/s/submit-now. Please also include the statement “There was no additional external funding received for this study.” in your updated Funding Statement. Please include your amended Funding Statement within your cover letter. We will change the online submission form on your behalf.

Response: Thanks for your kind reminder. We have provided the following amended Funding Statement:

“This work was supported by the Hubei Province Key Research and Development Special Project of Science and Technology Innovation Plan (No. 2023BAB087), the Wuhan Key Research and Development Projects (No. 2023010402010614), the Central Government Guides Local Funds for Science and Technology Development (No. ZYYD2024QY08) and the Wuhan Knowledge Innovation Special Dawn Project (No. 2023010201020465). Further support was provided by the open competition project for selecting the best candidates, Wuhan East Lake High-tech Development Zone (No. 2024KJB328) and the Fund for Research Platform of South-Central Minzu University (No. CZQ24011). There was no additional external funding received for this study.”

4. We note that your Data Availability Statement is currently as follows: [All relevant data are within the manuscript and its Supporting Information files.] Please confirm at this time whether or not your submission contains all raw data required to replicate the results of your study. Authors must share the “minimal data set” for their submission. PLOS defines the minimal data set to consist of the data required to replicate all study findings reported in the article, as well as related metadata and methods (https://journals.

plos.org/plosone/s/data-availability#loc-minimal-data-set-definition).

For example, authors should submit the following data:

- The values behind the means, standard deviations and other measures reported;

- The values used to build graphs;

- The points extracted from images for analysis.

Authors do not need to submit their entire data set if only a portion of the data was used in the reported study. If your submission does not contain these data, please either upload them as Supporting Information files or deposit them to a stable, public repository and provide us with the relevant URLs, DOIs, or accession numbers. For a list of recommended repositories, please see https://journals.plos.org/plosone/s/recom

mended-repositories. If there are ethical or legal restrictions on sharing a de-identified data set, please explain them in detail (e.g., data contain potentially sensitive information, data are owned by a third-party organization, etc.) and who has imposed them (e.g., an ethics committee). Please also provide contact information for a data access committee, ethics committee, or other institutional body to which data requests may be sent. If data are owned by a third party, please indicate how others may request data access.

Response: Thanks for your kind reminder. We have uploaded the minimal data set of the experimental results to the GitHub repository. The link is: [[https://github.com/cherr
ycsw/Video_Swin_CLSTM/tree/main/minimal_data_set](https://github.com/cherrycsw/Video_Swin_CLSTM/tree/main/minimal_data_set)]. All other relevant data are within the manuscript and its Supporting Information files.

**Reviewers' comments:**

***Reviewer #1:***

1. Abstract - Well-defined problem, but no evidence of results/quantitative values to support your claims, consider adding accuracy values. Some sentences need clarification since it does not fully represent the paper. For instance, “surpasses existing Swin Transformer variants” - the authors should give statistical significance values or comparative studies.

Response: We sincerely appreciate the reviewer's constructive feedback. In the “Abstract” section of revised manuscript (Page 2, Lines 26-40, highlighted with red), we have incorporated detailed statistical significance values and comparative analyses to substantiate our claims, as follows:

(1) Performance Improvement

Experiments on the UCF-101 dataset demonstrate that our model achieves mean Top-1/Top-5 accuracies of 92.8% and 99.4%, which are 3.2% and 2.0% higher than those of the baseline model (89.6% and 97.4%, respectively), These results are statistically significant (all experiments were repeated 5 times under identical configurations, with randomness controlled through distinct random seeds (0-4)).

(2) Component-wise Ablation Studies
    a) Optical flow fusion contributes +2.6% mean Top-1 accuracy (89.6%→92.2%).
    b) ConvLSTM module placement optimization provides +1.9% Top-1 gain (89.6%→91.5%).
    c) Custom ImageNet-1K-LSTM pretraining improves accuracy by +2.7% Top-1 (89.8%→92.5%)
(3) Computational Efficiency

Our optical flow fusion strategy reduces average computational costs by 3.3% compared to non-optical-flow architectures at peak performance levels.

The full experimental details are further expanded in the “Experiments” section (Table 1) and the “Ablation Study” section (Table 7) of the revised manuscript. Here, we also show the results of Tables 1 and 7 as follows:

2. Introduction - This section explains the significance of HAR in several domains, however, it gives too much information, perhaps consider reducing the size by turning into more concise paragraphs. The topics go over without an actual transition between paragraphs, please add words that link one paragraph to the next.

Response: Thanks for your valuable suggestions. In the “Introduction” section of revised manuscript (Pages 5-6, Lines 91-126 and 135-142, highlighted with red), we have carefully revised the original corresponding content to address your concerns with three key improvements:

(1) **Condensed Content**: Removed redundant domain examples (e.g., merged surveillance/sports/medical cases into a single application overview; moved the principles of some methods to the "Method" section)
(2) **Enhanced Coherence**: Added explicit transitions between paragraphs (e.g., "Despite significant advancements...", "Another critical challenge...", "To address these challenges...") to clarify the logical flow from applications to challenges to solutions.
(3) **Structured Contributions**: Replaced the original summary with a numbered list of innovations, directly linking each contribution to the identified challenges.

These revisions reduce the Introduction’s length nearly by 25% while improving readability. We believe the revised version now provides a clearer progression from problem motivation to technical solution.

3. Related Work - It is an intriguing section as provides a broad range of work in the literature, however, some studies are not well-described and quantitative information about the efficiency of the methods is not given. Also, please group similar studies - I often had to go back and forth to read similar work. I believe a big limitation that should be addressed is the need for more detailed information of the methods, it's difficult to know the depth of the literature gap without having the context of the field.

Response: We sincerely thank the reviewer for pointing out the important issue. To resolve the concerns you raised above, we have meticulously revised each of the four subsections within the "Related Work" section, "Based on Two-stream CNNs" (Pages 7-8, Lines 153-160 and 181-195, highlighted with red), "Based on RNNs" (Pages 9-10, Lines 217-232, highlighted with red), "Based on 3D CNNs"(Page 11, Lines 250-262, highlighted with red), and "Based on Transformer Models" (Page 12, Lines 282-296, highlighted with red). The main revisions are detailed below, using the first subsection “Based on Two-stream CNNs” as an example:

(1) Removed some original studies that are not relevant to the content of this research (e.g., Plizzari et al. concentrated on segmenting human body parts…), and some relevant references (with numbers in red font) have been added;
(2) Added quantitative metrics for some studies (e.g., 59.4% mean accuracy on HMDB51, at the cost of 6.2% performance loss);
(3) Grouping studies into noise robustness, long-term modeling, and efficiency-accuracy categories;
(4) Linked gaps (e.g., TSN’s fragmented context) to our methodology;
(5) Inserted transitional sentences (e.g., "These limitations, particularly fragmented temporal modeling and cross-domain degradation, motivate the integration of RNNs to...") to logically connect baselines and highlight research gaps.

The modifications to other subsections (“Based on RNNs”, “Based on 3D CNNs” and “Based on Transformer Models”) of the “Related Work” section are similar to the above works, aiming to ensure that the content is more streamlined, closely related to the research questions, and in-depth. We have also provided a detailed list of the references added to or removed from each subsection of the Related Work on the final appendix page.

4. Methodology - The architecture, as well as other information of the models are well-formulated and the authors included diagrams to facilitate the understanding. Some improvements I noticed are: 1) please focus on integrating more the equations, they are everywhere but without proper description. Also, please provide sources of each method, and potential work where I can replicate your method.

Response: Thank you for your insightful suggestions. We have carefully revised the methodology to enhance clarity, integrate equations with proper context, and ensure reproducibility. Below is a summary of key revisions aligned with your feedback, including explicit references for all critical components:

(1) Equation Integration and Explanation

In lines 373 to 386 (Pages 16-17) of revised manuscript “Method” section, we have integrated the calculation of the 3D SW-MSA module in Eq (1) with the formula of the ConvLSTM module, which makes the embedding of our ConvLSTM module more well-founded. Additionally, we provided a detailed explanation and description of the integrated formula, making it easier for readers to reproduce our method. The integrated Eq (3) is shown as follows:

(2) Clarification of Model Components

In lines 338 to 343 (Page 14) of revised manuscript “Method” section, we have reinterpreted the structure and its principle of the first unit of the Video Swin-CLSTM Transformer Block. This is to enable readers to better understand the working principle of the Video Swin-CLSTM Transformer Block and to reproduce this method.

(3) Reproducibility Enhancements

We have added explicit references or sources for all core methods (e.g., classification header [27], Multilayer Perceptron (MLP) module [30], Layer Normalization (LN) [31], TVL1 algorithm [34], LSTM [32], ConvLSTM [8], Video Swin Transformer [13]). The detailed references are as follows:

[8] Shi X, Chen Z, Wang H, Yeung DY, Wong WK, Woo Wc. Convolutional LSTM network: A machine learning approach for precipitation nowcasting. Advances in neural information processing systems. 2015;28.

[13] Liu Z, Ning J, Cao Y, Wei Y, Zhang Z, Lin S, et al. Video swin transformer. In: Proceedings of the IEEE/CVF conference on computer vision and pattern recognition; 2022. p. 3202–3211.

[27] Arnab A, Dehghani M, Heigold G, Sun C, Luˇci´c M, Schmid C. Vivit: A video vision transformer. In: Proceedings of the IEEE/CVF international conference on computer vision; 2021. p. 6836–6846.

[30] Dosovitskiy A, Beyer L, Kolesnikov A, Weissenborn D, Zhai X, Unterthiner T, et al. An image is worth 16x16 words: Transformers for image recognition at scale. arXiv preprint arXiv:201011929. 2020;

[31] Xiong R, Yang Y, He D, Zheng K, Zheng S, Xing C, et al. On layer normalization in the transformer architecture. In: International conference on machine learning. PMLR; 2020. p. 10524–10533.

[32] Vennerød CB, Kjærran A, Bugge ES. Long short-term memory RNN. arXiv preprint arXiv:210506756. 2021;.

[34] Fan L, Huang W, Gan C, Ermon S, Gong B, Huang J. End-to-end learning of motion representation for video understanding. In: Proceedings of the IEEE conference on computer vision and pattern recognition; 2018. p. 6016–6025.

(4) Structural and Diagrammatic Improvements
a) We have moved the optical flow stacking method from the "Sparse Sampling and Information Fusion" subsection under the "Method" section to the "Optical Flow Extraction" subsection, and then re-established a new subsection named "Optical Flow Extraction and Stacking", which makes the structure of the paper more reasonable. (Lines 409-411, Page 18)
b) After careful inspection, we found that in the original Fig 2 (Page 15, Lines 354-356, highlighted with red), neither the forget gate F_t_ nor the input gate I_t_ of the ConvLSTM architecture received the previous memory cell C_t-1_ as an input. Here, we have revised Fig 2, and the current version is the latest one. We have re-uploaded the updated Fig 2 to the system. The revised Fig 2 is below:

**Fig 2**. **The details of Video Swin-CLSTM transformer block.** (a) Illustration of two successive Video Swin-CLSTM Transformer block units; (b) Overview of Convolutional LSTM.

5. Experiments - I personally enjoyed reading this section since it's well-structured and has a good explanation of the training and testing setup. However, it is unclear how the hyperparameters were chosen, and I believe it also need better detail about the baseline used for comparison.

Response: Thanks for your valuable suggestions. We have carefully revised the “Experiments” section to address your concerns regarding hyperparameter selection and baseline comparison. The revised content in the revised manuscript pages 21-22 lines 489-499, and the key improvements are as follows:

(1) Training Epochs: utilized an AdamW optimizer over 50 epochs (vs. Video Swin-T's 30 epochs) to more comprehensively optimize the model's parameters
(2) Batch Size: Adjusted from 64 to 16 (-75%) due to lower GPU memory compared to the baseline model experiments and the additional computational overhead brought by the ConvLSTM module.
(3) Backbone Learning Rate: Scaled down by 0.1× (from 0.001 to 0.0001) to preserve pre-trained feature stability while allowing new ConvLSTM layers to train at full rate (0.001).
(4) Temporal Stride: Changed from 2 to 1 to capture fine-grained motion patterns.

These modifications ensure both methodological transparency and fair comparison. We appreciate your insightful comments that helped strengthen our experimental rigor.

6. Results & Ablation Study - I had problem understanding at first the Top 1 and Top 5 accuracy values, as it is not intuitive. Please give some thought in how to enhance this part. In Table 1, the Param information was not defined by the caption, so please do so. Also, this is an IMPORTANT point, please provide statistical evaluation of your method in order to claim significant effects. As the paper currently stands, all your findings can be due to chance of reaching better results. This has a big impact on your result (and due to this topic I'm suggesting major reviews).

Response: We sincerely thank the reviewer for pointing out these important issues.

(1) Regarding the first issue raised by the reviewer—Top-1 and Top-5 metrics not being intuitive—we have added a background explanation in the first paragraph of the revised “Results” section (Page 22, Lines 514-517, highlighted with red) to clarify the meaning and significance of these evaluation metrics. Additionally, to better illustrate our model's effectiveness, we have incorporated Inference (ms/frame) and Memory (GB) metrics along with corresponding experimental data in Table 1 (Page 23, Lines 531-535, highlighted with red) to improve result comparability. Furthermore, in Table 4 (Page 29, Lines 655-657), which presents the ablation study on the embedding position of the ConvLSTM module, we have included the Gradient Variance metric and its corresponding experimental results to better explain why embedding the ConvLSTM module between 3D SW-MSA and the residual connection achieves the best performance.
(2) Regarding the second issue—the lack of a definition for the "Param" metric—we have added an explanation for this metric in the caption of Table 1 (Page 23, Line 534, highlighted with red).
(3) Regarding the third issue—the absence of statistical evaluation for our proposed method—we would like to clarify that all experimental results in the original manuscript were obtained by running each experiment five times under identical configurations (including dataset splits, hyperparameters, and hardware conditions), with different random seeds (0 to 4) used to control variability. However, this detail was not explicitly stated in the original text. To emphasize the statistical evaluation of our experimental results, we have added a description of our statistical evaluation approach in the first paragraph of the revised “Results” section (Page 22, Lines 517-520, highlighted with red) and included the statistically evaluated results in Table 1.

The revised first paragraph of the “Results” section, along with the updated Tables 1 and 4, are presented below:
 “In action recognition tasks, model performance is quantified by Top-1 and Top-5 accuracy (%). Top-1 accuracy measures the proportion of test samples where the model’s highest-confidence prediction matches the ground-truth label, while Top-5 accuracy evaluates whether the true label is within the top five predictions, which is crucial for distinguishing ambiguous or fine-grained actions. To ensure rigorous comparisons, all experiments were repeated 5 times under identical configurations (dataset splits, hyperparameters, hardware), with randomness controlled through distinct random seeds (0-4) governing parameter initialization, data augmentation, and batch ordering. This multi-run protocol isolates the impact of stochastic factors, ensuring reported results reflect consistent model capability.”

7. Conclusion & Future Work - summarized properly the contributions. However, future work should be better aligned as only broader ideas were given. Also, in the perfect world, what would be your next experiment if you had more data or perhaps more complementary data?

Response: We sincerely thank the reviewer for pointing out the important issue. We have carefully revised the “Conclusion and Future Work” section to address your concerns regarding the next steps for experiments on challenging datasets. The revised content in the revised manuscript page 35 lines 791-811 as follows:

“In future work, we aim to investigate and validate the generalizability of the proposed innovations on more extensive and challenging datasets. Although the proposed model demonstrates robust performance under standard conditions, its effectiveness may diminish under heavy occlusions or extreme lighting due to its reliance on spatially continuous optical flow and fixed-depth temporal modeling. To address these limitations, we plan to investigate the integration of multi-modal inputs (e.g., audio cues for occluded scenes and infrared imaging for low-light conditions) to compensate for compromised visual data. Moreover, we will propose developing adaptive ConvLSTM architectures that dynamically adjust their layer depth based on scene complexity, thereby enabling tailored temporal modeling for a wider range of challenges.”

“While the ConvLSTM-enhanced model excels at local temporal dynamics, it struggles with long-range semantic integration in multi-modal videos. In the next experiment, we will integrate a learnable external memory module to enhance long-term multi-modal reasoning. This module will maintain dedicated memory banks for various modal data streams within a shared semantic space, enabling cross-modal retrieval through content-based addressing (e.g., using audio queries like ‘glass breaking’ to locate relevant video segments). Adaptive gates will fuse ConvLSTM's local temporal features with global memory states, combining short-term dynamics with persistent semantics. This approach could reduce information loss in extended videos while offering interpretable memory visualization.”

“In terms of reducing computational overhead, optical flow does lower the cost; however, its approach of pixel-based reduction is inherently limited. Therefore, we plan to incorporate a more efficient tube-based sparse sampling method into our model. By defining tubes of various sizes and depths, the model can learn feature information at different levels in the input video (e.g., fine-grained, short-term actions and coarse-grained, long-term actions), while significantly reducing computational overhead and maintaining a lightweight architecture. These improvements are expected to be implemented and validated in future experiments, ultimately advancing video-based human action recognition and expanding its practical applications across various domains.”

***Reviewer #2:***

1. This paper could benefit from a more concise presentation of the related work section, as it currently feels lengthy and could be streamlined to focus more on the most relevant studies.

Response: We sincerely thank the reviewer for pointing out the important issue. To resolve the concerns you raised above, we have meticulously revised each of the four subsections within the "Related Work" section, "Based on Two-stream CNNs" (Pages 7-8, Lines 153-160 and 181-195, highlighted with red), "Based on RNNs" (Pages 9-10, Lines 217-232, highlighted with red), "Based on 3D CNNs"(Page 11, Lines 250-262, highlighted with red), and "Based on Transformer Models" (Page 12, Lines 282-296, highlighted with red). The main revisions are detailed below, using the first subsection “Based on Two-stream CNNs” as an example:

(1) Removed some original studies that are not relevant to the content of this research (e.g., Plizzari et al. concentrated on segmenting human body parts…), and some relevant references (with numbers in red font) have been added;
(2) Added quantitative metrics for some studies (e.g., 59.4% mean accuracy on HMDB51, at the cost of 6.2% performance loss);
(3) Grouping studies into noise robustness, long-term modeling, and efficiency-accuracy categories;
(4) Linked gaps (e.g., TSN’s fragmented context) to our methodology;
(5) Inserted transitional sentences (e.g., " These limitations, particularly fragmented temporal modeling and cross-domain degradation, motivate the integration of RNNs to...") to logically connect baselines and highlight research gaps.

The modifications to other subsections (“Based on RNNs”, “Based on 3D CNNs” and “Based on Transformer Models”) of the “Related Work” section are similar to the above works, aiming to ensure that the content is more streamlined, closely related to the research questions, and in-depth. We have also provided a detailed list of the references added to or removed from each subsection of the Related Work on the final appendix page.

2. Provide a detailed analysis of how optical flow impacts performance in different motion scenarios (e.g., fast motions, occlusions).

Response: Thank you for your insightful suggestions. To address your concern, we have mainly carried out the following two aspects of work:

(1) In the “Method” section of the revised manuscript (Page 17, Lines 388-395, highlighted with red), we have analyzed the underlying principles of optical flow and explained why it can overcome the impact of environmental changes on human action recognition, thereby providing a theoretical basis for the subsequent work.
(2) In the “Ablation Study” section of the revised manuscript (Pages 26-28, Lines 607-644, highlighted with red), we have included a set of ablation experiments to investigate the robustness of optical flow. First, we extracted sub-datasets representing different scenarios (fast motion, occlusive motion, subtle motion) from the UCF-101 dataset. Then, both the baseline model and the new model were trained and validated on these sub-datasets, with the experimental results summarized in Table 3. We further analyzed these results and concluded that optical flow can effectively mitigate the impact of environmental changes on model accuracy and significantly assist the model in recognizing short-term actions. To better understand the interference robustness of optical flow, we have also visualized the motion features under different scenarios, as shown in Fig. 6.

Fig 6. **Visualization of optical flow under different motion scenarios.** (a) Fast Motion (*BasketballShooting*); (b) Occlusive Motion (*SoccerJuggling*); (c) Subtle Motion (*ApplyEyeMakeup*).

3. Emphasize how the proposed model advances the state-of-the-art in HAR compared to existing Transformer-based models.

Response: We sincerely appreciate the reviewer’s valuable comments. Our final experimental results were compared with Swin Transformer-based video models, and we mainly emphasized the superiority of our model over existing Swin Transformer-based video models from the following two aspects.

(1) In the “Abstract” section of revised manuscript (Pages 2-3, Lines 33-40, highlighted with red), we have incorporated detailed statistical significance values and comparative analyses to substantiate our claims, as follows:

“Experiments on the UCF-101 dataset demonstrate that our model achieves mean Top-1/Top-5 accuracies of 92.8% and 99.4%, which are 3.2% and 2.0% higher than those of the baseline model (89.6% and 97.4%, respectively), while the computational cost is reduced by an average of 3.3% at peak performance compared to models without optical flow. Ablation studies further validate the effectiveness of our model’s crucial components, with the integration of optical flow and the embedding of ConvLSTM modules yielding maximum improvements in mean Top-1 accuracy of 2.6% and 1.9%, respectively. Notably, employing our custom ImageNet-1K-LSTM pre-training model results in a maximum increase of 2.7% in mean Top-1 accuracy compared to traditional ImageNet-1K pre-training model. These experimental results indicate that our model offers certain advantages over other Swin Transformer-based methods for video HAR tasks.”

(2) In the comparative analysis (Pages 22-24, Lines 521-564) of the “Result” secton of the revised manuscript, we have evaluated our proposed model against other Swin Transformer-based video models from three perspectives: the use of a custom pre-trained model, the incorporation of optical flow information, and the embedding of the ConvLSTM module. Additionally, we added the Inference (ms/frame) and Memory (GB) metrics to assess our model's efficiency, as shown in Table 1. When analyzing the experimental results, we compared multiple evaluation metrics (Top-1, Top-5, FLOPs, Param, Inference, and Memory) against existing Swin Transformer-based video models. The final conclusion was that our model achieved the highest accuracy while maintaining a reasonable computational cost.

4. Justify why ConvLSTM performs best between the 3D SW-MSA module and the residual connection.

Response: We sincerely appreciate this insightful question. After the “Position of the ConvLSTM Module” subsection of revised manuscript (Pages 29-30, Lines 668-685, highlighted with red), we have added a theoretical analysis to rigorously justify the optimal ConvLSTM placement:

(1) Information flow analysis

Placing ConvLSTM between 3D SW-MSA and residual connections enables complementary global-temporal (via SW-MSA) and local-spatial (via residual) feature learning, formalized as:

(2) Gradient stability validation

We introduced a gradient variance metric (details in Eq 5), showing (Table 4) our design reduces variance by 71% and 63% compared to pre-SW-MSA placement (0.11 vs. 0.38) and post-residual placement (0.11 vs. 0.29), respectively.

These additions systematically validate that the optimal positioning balances global-temporal modeling and local-spatial preservation, while ensuring stable gradient flow. We thank the reviewer for prompting this deeper analysis, which strengthens our methodological contribution.

5. Explore the effect of stacking multiple ConvLSTM layers for potential performance improvements.

Response: We thank the reviewer for raising this important comments. We have added “Investigation of Multi-layer ConvLSTM Stacking” subsection in the “Ablation Study” section of the revised manuscript (Pages 32-33, Lines 724-745, highlighted with red), and explored multi-layer ConvLSTM stacking via intra-stage (Stage2+4) and cross-stage (Stage1+2+4) configurations.

Experimental results (Table 6) and Fig 7 show that stacking ConvLSTM layers consistently degrades model accuracy and efficiency. This phenomenon likely stem from three limitations: deep layers oversmooth temporal features, erasing high-frequency motion details essential for fine-grained actions; low-level stages (e.g., Stage1) face spatial-temporal conflicts as they prioritize static semantics over dynamic patterns; and optimization instability arises from vanishing gradients and redundant parameters that disrupt temporal signal propagation.
 These results validate our baseline’s efficiency: two-stage ConvLSTM distribution optimally balances temporal depth and computational cost. Due to limited GPU computing resources, we will discuss potential solutions (e.g., more layer/stage stacking (num_layer > 3) and dynamic layer pruning) as future work.

**Fig 7**. **Accuracy vs FLOPs/Memory Trade-offs in ConvLSTM Layer Stacking**.

6. Discuss potential limitations when applied to more challenging datasets with occlusions or extremelighting conditions.

Response: Thanks for your valuable suggestions. We have further discussed the potential limitations of our approach when applied to more challenging datasets featuring occlusions or extreme lighting conditions, and have highlighted the changes on page 35 (lines 791-797) of the revised manuscript as follows:

“In future work, we aim to investigate and validate the generalizability of the proposed innovations on more extensive and challenging datasets. Although the proposed model demonstrates robust performance under standard conditions, its effectiveness may diminish under heavy occlusions or extreme lighting due to its reliance on spatially continuous optical flow and fixed-depth temporal modeling. To address these limitations, we plan to investigate the integration of multi-modal inputs (e.g., audio cues for occluded scenes and infrared imaging for low-light conditions) to compensate for compromised visual data. Moreover, we will propose developing adaptive ConvLSTM architectures that dynamically adjust their layer depth based on scene complexity, thereby enabling tailored temporal modeling for a wider range of challenges.”

7. Provide deeper insights into the feature interactions and performance impact of each model component in the ablation studies.

Response: We appreciate the reviewer’s comments and thoughtful consideration of our study. We have provided deeper insights into the feature interactions and performance impact of each model component in our ablation studies, primarily in the following aspects:

(1) For the optical flow information introduced into the model, we not only explored the impact of different optical flow proportions as input on model performance in the “Sparse Sampling and Information Fusion” subsection of the revised manuscript, but also conducted a set of ablation experiments (the “Robustness of Optical Flow” subsection) to assess the robustness of optical flow. Furthermore, we analyzed how optical flow influences model performance across different scenarios (fast motion, occluded motion, and subtle motion) and attempted to elucidate the underlying causes of the observed results.
(2) For the ConvLSTM module embedded in the model, we conducted a more in-depth theoretical analysis (Pages 29-30, Lines 668-685, highlighted with red) of its optimal embedding position from two perspectives: information flow analysis and gradient propagation stability. Additionally, in newly added ablation experiments (the “Investigation of Multi-layer ConvLSTM Stacking” subsection), we explored the effect of stacking multiple ConvLSTM layers to assess potential performance improvements. The experimental results validated our baseline’s efficiency: a two-stage ConvLSTM distribution optimally balanced temporal depth and computational cost.
(3) For the custom pre-trained model, we have provided a more profound and detailed explanation (Pages 33-34, Lines 756-760, highlighted with red) of how using a custom pre-trained model can enhance the performance of the model.

8. Table 1 provides a good comparison of different models, but it could be enhanced by including additional metrics such as inference time or memory usage.

Response: Thanks for your valuable suggestions. In the “Result” section of revised manuscript (Page 23, Lines 531-535, highlighted with red), we have added the Inference (ms/frame) and Memory (GB) metrics to Table 1 to enhance the rigor of the experiments and improve the comparability of the results. The revised Table 1 is shown below:

9. In Figure 1 add more annotations or labels to clarify the flow of data.

Response: We sincerely appreciate the reviewer’s valuable comments. We have modified the original Fig 1 by adding more annotations and labels and updating the caption to clarify the flow of data, we highlighted the changes on page 14 lines 327-332 of the revised manuscript as follows.

Fig 1. The architecture of Video Swin-CLSTM Transformer. It is composed of four stages, with the number of blocks in each stage being 2, 2, 6, and 2, respectively. In Stage 2 and Stage 4, the core modules are Video Swin-CLSTM Transformer blocks with spatiotemporal attention and memory units, while the remaining stages utilize Video Swin Transformer blocks with spatiotemporal attention only. The description above the figure illustrates the changes in the model data dimensions throughout the process.

10. Provide a comparison of the model's efficiency in terms of FLOPs, inference time, and memory usage.

Response: Thanks for your valuable suggestions. In the revised manuscript (Page 23, Lines 531-535, highlighted with red), we have added the Inference (ms/frame) and Memory (GB) metrics to Table 1 and incorporated an analysis of these two metrics in the experimental results (Pages 23-24, Lines 536-564) to enhance the rigor of the study and improve the comparability of the results. Additionally, we included these two metrics in Table 2 (Page 25, Lines 591-593) and modified Fig5 (Page 26, Lines 605-606, highlighted with red) by replacing the Param metric in (b) with the Inference metric, which better reflects the reduction in model resource consumption. The revised Table 2 and Fig 5 are shown below:

Fig 5. Effect of the proportion of optical flow input on model accuracy (a) and computational overhead (b).

Lastly, the reviewers provided constructive feedback on the structure and some minor expression problems of our paper, offering reasonable suggestions for improvement. We have also made careful revisions on a case-by-case basis.

We confirm that neither the manuscript nor any parts of its content are currently under

consideration or published in another journal. All authors have approved the manuscript and agree with its submission to PLOS ONE.

If you have any other points or suggestions regarding the paper that you would like us to address, please feel free to provide further guidance. We are eager to meet your expectations and deliver a manuscript that meets the highest standards.

Thank you for your constructive feedback.

Sincerely,

Zheng Ye (corresponding author)

[yezheng@scuec.edu.cn](mailto:yezheng@scuec.edu.cn)

March 28, 2025

**Appendix**

- Based on Two-stream CNNs

Bilen H, Fernando B, Gavves E, Vedaldi A, Gould S. Dynamic image networks for action recognition. In: Proceedings of the IEEE conference on computer vision and pattern recognition; 2016. p. 3034–3042. (added)

Karpathy A, Toderici G, Shetty S, Leung T, Sukthankar R, Fei-Fei L. Large-scale video classification with convolutional neural networks. In: Proceedings of the IEEE conference on Computer Vision and Pattern Recognition; 2014. p. 1725–1732. (removed)

Plizzari C, Cannici M, Matteucci M. Skeleton-based action recognition via spatial and temporal transformer networks. Computer Vision and Image Understanding. 2021; 208:103219. (removed)

Girdhar R, Ramanan D, Gupta A, Sivic J, Russell B. Actionvlad: Learning spatio-temporal aggregation for action classification. In: Proceedings of the IEEE conference on computer vision and pattern recognition; 2017. p. 971–980. (removed)

Zong M, Wang R, Chen X, Chen Z, Gong Y. Motion saliency based multi-stream multiplier ResNets for action recognition. Image and Vision Computing. 2021; 107:104108. (removed)

Liu Z, Cheng J, Liu L, Ren Z, Zhang Q, Song C. Dual-stream cross-modality fusion transformer for RGB-D action recognition. Knowledge-Based Systems. 2022; 255:109741. (removed)

Chen Z, Li S, Yang B, Li Q, Liu H. Multi-scale spatial temporal graph convolutional network for skeleton-based action recognition. In: Proceedings of the AAAI conference on artificial intelligence. vol. 35; 2021. p. 1113–1122. (removed)

Piergiovanni A, Ryoo MS. Representation flow for action recognition. In: Proceedings of the IEEE/CVF conference on computer vision and pattern recognition; 2019. p. 9945–9953. (removed)

- Based on RNNs

Ballas N, Yao L, Pal C, Courville A. Delving deeper into convolutional networks for learning video representations. arXiv preprint arXiv:151106432. 2015;. (added)

Campos V, Jou B, Gir´o-i Nieto X, Torres J, Chang SF. Skip rnn: Learning to skip state updates in recurrent neural networks. arXiv preprint arXiv:170806834. 2017;. (added)

Yue-Hei Ng J, Hausknecht M, Vijayanarasimhan S, Vinyals O, Monga R, Toderici G. Beyond short snippets: Deep networks for video classification. In: Proceedings of the IEEE conference on computer vision and pattern recognition; 2015. p. 4694–4702. (removed)

Wu Z, Xiong C, Jiang YG, Davis LS. Liteeval: A coarse-to-fine framework for resource efficient video recognition. Advances in neural information processing systems. 2019;32. (removed)

Majd M, Safabakhsh R. Correlational convolutional LSTM for human action recognition. Neurocomputing. 2020; 396:224–229. (removed)

Ge H, Yan Z, Yu W, Sun L. An attention mechanism based convolutional LSTM network for video action recognition. Multimedia Tools and Applications. 2019; 78:20533–20556. (removed)

Sudhakaran S, Escalera S, Lanz O. Lsta: Long short-term attention for egocentric action recognition. In: Proceedings of the IEEE/CVF conference on computer vision and pattern recognition; 2019. p. 9954–9963. (removed)

Girdhar R, Ramanan D. Attentional pooling for action recognition. Advances in neural information processing systems. 2017;30. (removed)

Meng L, Zhao B, Chang B, Huang G, Sun W, Tung F, et al. Interpretable spatio-temporal attention for video action recognition. In: Proceedings of the IEEE/CVF international conference on computer vision workshops; 2019. (removed)

Wu Z, Xiong C, Ma CY, Socher R, Davis LS. Adaframe: Adaptive frame selection for fast video recognition. In: Proceedings of the IEEE/CVF Conference on Computer Vision and Pattern Recognition; 2019. p. 1278–1287. (removed)

Liu Z, Li Z, Wang R, Zong M, Ji W. Spatiotemporal saliency-based multi-stream networks with attention-aware LSTM for action recognition. Neural Computing and Applications. 2020;32(18):14593–14602. (removed)

Li Z, Gavrilyuk K, Gavves E, Jain M, Snoek CG. Videolstm convolves, attends and flows for action recognition. Computer Vision and Image Understanding. 2018; 166:41–50. (removed)

Yang J, Dong X, Liu L, Zhang C, Shen J, Yu D. Recurring the transformer for video action recognition. In: Proceedings of the IEEE/CVF Conference on Computer Vision and Pattern Recognition; 2022. p. 14063–14073. (removed)

Shi Y, Tian Y, Wang Y, Zeng W, Huang T. Learning long-term dependencies for action recognition with a biologically-inspired deep network. In: Proceedings of the IEEE International Conference on Computer Vision; 2017. p. 716–725. (removed)

Dwibedi D, Sermanet P, Tompson J. Temporal reasoning in videos using convolutional gated recurrent units. In: Proceedings of the IEEE Conference on Computer Vision and Pattern Recognition Workshops; 2018. p. 1111–1116. (removed)

Kim PS, Lee DG, Lee SW. Discriminative context learning with gated recurrent unit for group activity recognition. Pattern Recognition. 2018;76:149–161. (removed)

Cho K. Learning phrase representations using RNN encoder-decoder for statistical machine translation. arXiv preprint arXiv:14061078. 2014. (removed)

Chung J, Gulcehre C, Cho K, Bengio Y. Empirical evaluation of gated recurrent neural networks on sequence modeling. arXiv preprint arXiv:14123555. 2014. (removed)

- Based on 3D CNNs

Tran D, Wang H, Torresani L, Ray J, LeCun Y, Paluri M. A closer look at spatiotemporal convolutions for action recognition. In: Proceedings of the IEEE conference on Computer Vision and Pattern Recognition; 2018. p. 6450–6459. (added)

Diba A, Fayyaz M, Sharma V, Karami AH, Arzani MM, Yousefzadeh R, et al. Temporal 3d convnets: New architecture and transfer learning for video classification. arXiv preprint arXiv:171108200. 2017. (removed)

Varol G, Laptev I, Schmid C. Long-term temporal convolutions for action recognition. IEEE transactions on pattern analysis and machine intelligence. 2017;40(6):1510–1517. (removed)

Wang X, Gao L, Wang P, Sun X, Liu X. Two-stream 3-d convnet fusion for action recognition in videos with arbitrary size and length. IEEE Transactions on Multimedia. 2017;20(3):634–644. (removed)

Feichtenhofer C, Fan H, Malik J, He K. Slowfast networks for video recognition. In: Proceedings of the IEEE/CVF international conference on computer vision; 2019. p. 6202–6211. (removed)

Qiu Z, Yao T, Mei T. Learning spatio-temporal representation with pseudo-3d residual networks. In: proceedings of the IEEE International Conference on Computer Vision; 2017. p. 5533–5541. (removed)

- Based on Transformer Models

In this part, we did not add or delete any references in the original manuscript.
