# Supplementary material for: Video swin-CLSTM transformer: Enhancing human action recognition with optical flow and long-term dependencies
Source: PLoS One. 2025 Jul 7;20(7):e0327717. doi: 10.1371/journal.pone.0327717 (PMC12233284; doi:10.1371/journal.pone.0327717)
Supplement: Attachment — Submitted filename: Response_to_Reviewers_auresp_2.docx [file pone.0327717.s003.docx]

**Response to Reviewers**

Dear Editor and Reviewers:

Thank you for your continued review of our manuscript entitled “Video Swin-CLSTM Transformer: Enhancing Human Action Recognition with Optical Flow and Long-Term Dependencies” (**PONE-D-24-45158R1**). We sincerely appreciate the further comments and suggestions provided by the editor. In this minor revision, we have carefully addressed the editor’s concerns point by point in the response and revised the manuscript accordingly. We hope that the revised manuscript now meets your expectations and is accepted for publication.

**Journal requirements:**

1. Please review your reference list to ensure that it is complete and correct. If you have cited papers that have been retracted, please include the rationale for doing so in the manuscript text, or remove these references and replace them with relevant current references. Any changes to the reference list should be mentioned in the rebuttal letter that accompanies your revised manuscript. If you need to cite a retracted article, indicate the article’s retracted status in the References list and also include a citation and full reference for the retraction notice.

Response: Thank you for your kind reminder. After our repeated and careful review, we confirm that none of the references cited in this paper have been retracted. However, we have identified the following two issues:

(1) Some author names in the references appear garbled. For example, in reference [12], the author is listed as “Kr¨ahenb¨uhl P”; in reference [14], as “G¨uney F”; in reference [24], as “Gir´o-i Nieto X”; and in reference [27], as “Luˇci´c M”. The revised references, with corrected author names, are highlighted in red below the original references in the revised manuscript. (Pages 29-30, Lines 658, 665, 688, 696).
(2) The in-text citations do not use the cross-referencing format. Therefore, we have modified all in-text references to use the cross-reference format, facilitating readers’ quick access to the corresponding references.

We hope that the revised references now meet the journal’s formatting requirements.

**Additional Editor Comments:**

1. The text on line 576 of the reviewed PDF is very strange -- "In Sec Sparse Sampling and Information Fusion Sparse Sampling and Information Fusion Sparse Sampling and

Information Fusion Sparse Sampling and Information Fusion, we investigated ". Please fix it.

Response: We sincerely thank the editor for careful reading. We feel sorry for our carelessness about the appearance of the strange text. Due to the use of cross-referencing during the revision process, the section title “Sparse Sampling and Information Fusion” was inadvertently repeated. We have corrected it on page 18, line 395 of the revised manuscript as follows.

“In Sec Sparse Sampling and Information Fusion, we investigated…”

2. In lines 291, 426, 543, 608, please avoid positional words such as "above" in the text -- use the section name or figure name (the "above" in line 184 is OK).

Response: We sincerely appreciate the editor’s detailed review and valuable feedback. To address the concern regarding the use of positional words such as “above,” we have revised the manuscript as follows:

(1) On page 10, lines 219-220, we replaced "Above" with "Eq (2)".
(2) On page 19, line 427, we replaced "Above" with "In Sec Sparse Sampling Fusion Scheme".
(3) On page 25, line 545, we replaced "In the ablation experiments above" with "In Sec Position of the ConvLSTM Module and Sec Distribution of the Video Swin-CLSTM Block".

We have carefully reviewed the manuscript to ensure consistency in terminology and improve clarity. Thank you for your guidance.

Lastly, we sincerely appreciate the editor's detailed review and constructive feedback. The minor revisions requested have been carefully addressed, and all necessary corrections have been made to improve the clarity and accuracy of the manuscript.

If you have any other points or suggestions regarding the paper that you would like us to address, please feel free to provide further guidance. We are eager to meet your expectations and deliver a manuscript that meets the highest standards.

Thank you for your constructive feedback.

Sincerely,

Zheng Ye (corresponding author)

[yezheng@scuec.edu.cn](mailto:yezheng@scuec.edu.cn)

April 3, 2025
